# Risk factors for infectious enteritis after temporary stoma closure following colorectal cancer surgery: a retrospective cohort study

**DOI:** 10.1097/MS9.0000000000004981

**Published:** 2026-04-16

**Authors:** Reina Onaka, Hironori Fukuoka, Susumu Daibo, Hiroki Ohya, Kazuya Nakagawa, Mayumi Ozawa, Yusuke Saigusa, Hirotoshi Akiyama, Itaru Endo

**Affiliations:** a School of Medicine, Yokohama City University, Yokohama, Kanagawa, Japan; b Department of Gastroenterological Surgery, Yokohama City University Hospital, Yokohama, Kanagawa, Japan; c YCU Center for Novel and Exploratory Clinical Trials (Y-NEXT), Yokohama City University Hospital, Yokohama, Kanagawa, Japan; d Department of Gastroenterological Surgery, Yokohama City University Graduate School of Medicine, Yokohama, Kanagawa, Japan; e Department of Biostatistics, Yokohama City University Graduate School of Medicine, Yokohama, Kanagawa, Japan

**Keywords:** colorectal cancer, delayed stoma closure, infectious enteritis, logistic regression analysis, stoma closure

## Abstract

**Background:**

This study aimed to identify risk factors for infectious enteritis after temporary stoma closure in patients undergoing primary colorectal cancer surgery.

**Materials and Methods:**

A retrospective analysis of 201 patients who underwent stoma closure between 2009 and 2024 at our hospital was conducted using patient demographics, surgical details, and postoperative factors. Infectious enteritis was defined as Clavien–Dindo grade II or higher, based on clinical symptoms and treatment requirements. Statistical analysis was performed to identify significant differences (*P* < 0.05). Logistic regression was used to identify independent risk factors.

**Results:**

The incidence of infectious enteritis was 4%. Patients who developed infectious enteritis had significantly longer intervals between stoma creation and closure (274 vs 114 days, *P* = 0.002) and lower intraoperative bleeding volumes (0 vs 16 ml, *P* = 0.004). In exploratory regression analysis, a longer interval between stoma creation and closure was associated with the occurrence of infectious enteritis. Logistic regression identified the closure interval (per 100 days) as an independent risk factor (odds ratio: 1.574, 95% confidence interval: 1.138–2.411, *P* = 0.025).

**Conclusion:**

In this exploratory cohort, a longer interval between stoma creation and closure was associated with infectious enteritis after stoma closure. These findings require confirmation in larger studies and should be interpreted in the context of oncologic treatment requirements, anastomotic safety, and overall patient condition when considering the timing of stoma closure.

## Introduction

The number of patients with colorectal cancer is increasing worldwide, including in Japan. Anastomotic leakage is one of the most serious postoperative complications that occurs after surgery for colorectal cancer. To avoid this complication, a temporary stoma is sometimes created^[^1,2^]^, which is closed when the patient’s postoperative treatment and general condition stabilize. However, several complications have been reported after temporary stoma closure^[^3–5^]^, and intestinal inflammation is often encountered in clinical practice. Although complications after temporary stoma closure have been described, enteritis is not generally evaluated^[^3,5–7^]^ as no definition of postoperative enteritis has been established, and its symptoms are similar to those of low anterior resection syndrome (LARS)[8]. However, in some cases, clinicians provide treatment for suspected “infectious enteritis” after surgery. Therefore, this study aimed to clarify the risk factors for developing infectious enteritis during hospitalization after temporary stoma closure in patients undergoing colostomy after colorectal cancer surgery based on an analysis of real-world data.

HIGHLIGHTS

- We identified risk factors for enteritis after temporary stoma closure in colorectal cancer.
- The incidence of infectious enteritis was 4%.
- Infectious enteritis tended to occur more frequently with longer closure intervals.
- The closure interval (per 100 days) was associated with infectious enteritis.
- Further studies are needed to understand prevention and management strategies better.

## Methods

### Patients

Patients who underwent surgery at our hospital between March 2009 and July 2024 were included in this study. Patients with primary colorectal cancer who underwent surgery with anastomosis and stoma creation were eligible for inclusion in the study. Cases with concomitant inflammatory bowel disease and cases for which information was not available at the time of stoma closure were excluded. Among these patients, the data for those who underwent stoma closure were retrospectively reviewed.

The study protocol was approved by the Institutional Ethics Committee. The study was registered with the Japanese Clinical Trials Registry (https://www.umin.ac.jp/ctr/index.htm). As this was a retrospective study and the subjects’ information was treated as pseudonymized, only existing information from our facility was used. To provide participants with the opportunity to refuse participation, an opt-out method was employed, and the ethics committee approved the decision not to obtain written consent (Ethical Guidelines for Medical and Biological Research Involving Human Subjects). This study complies with the STROCSS guidelines[9], and no AI tools were used.

### Patient data

The following data were collected during the evaluation of infectious enteritis during the period from stoma creation to its closure and evaluation:

Patient background before stoma creation: age, sex, body mass index (BMI), prognostic nutritional index (PNI), American Society of Anesthesiologists physical status (ASA-PS), smoking history, gastrointestinal surgery history, and primary tumor site.

Information at the time of stoma creation: history of treatment before primary tumor surgery, stoma creation before primary tumor surgery, location of stoma, percentage of rectal resection, percentage of lateral pelvic lymph node dissection rate, final stage according to the eighth edition of the Union for International Cancer Control classification, rate of complications of Clavien–Dindo (CD) grade II or higher at the time of primary tumor surgery, rate of adjuvant chemotherapy between primary surgery and stoma closure, and treatment history after stoma creation (including cases where a stoma was created and treated before primary tumor surgery).

Information on stoma closure: Time from stoma creation to closure, surgery operation time, amount of bleeding, concomitant resection, maximum number of bowel movements per day within 1 week after surgery, number of days of perioperative antimicrobial administration, percentage of patients taking probiotics after surgery, and number of patients with detection of *Clostridioides difficile* enteritis toxin A/B.

### Definition of infectious enteritis in this study

Cases in which clinicians suspected infectious enteritis of CD grade II or higher based on abdominal and defecation symptoms, postoperative inflammatory values, imaging tests, and treatment requirements were defined as having infectious enteritis (IE group). One symptom of infectious enteritis is frequent bowel movements, which is also a symptom of LARS. In general, the use of antidiarrheal drugs is not recommended in cases diagnosed with infectious enteritis[10]. Therefore, in this study, patients for whom antidiarrheal drugs were administered had LARS, and they were not classified in the IE group. As frequent bowel movements and mild inflammatory changes can occur as part of the normal postoperative course following rectal cancer surgery, infectious enteritis in this study was diagnosed based on a comprehensive clinical assessment rather than a single finding.

### Incidence of infectious enteritis from the time of stoma creation to stoma closure

The time from stoma creation to closure was divided into six bins of 100 days each, and the incidence of infectious enteritis after stoma closure was calculated for each bin.

### Statistical analysis

All data were analyzed for manipulation and visualization in R[11] using packages available as part of tidyverse[12], including readr and ggplot[2], as well as tableone[13] and rms[14], respectively. Quantitative data were expressed as medians and interquartile range (in Supplemental Digital Content Tables 1–7, available at: http://links.lww.com/MS9/B120, when the sample size was limited to two participants, the data are reported as median [range] to reflect individual values). Similarly, when the sample size was limited to a single participant, the raw values were reported. Two groups were compared using Mann–Whitney *U* and Fisher’s exact tests, as appropriate. *P* < 0.05 was considered statistically significant. Multivariable logistic regression analyses were performed for the incidence of infectious enteritis. Because the number of infectious enteritis events was small (*n* = 8), the regression analyses were performed as exploratory analyses, and the results should be interpreted strictly as hypothesis-generating. Although traditional recommendations suggest a minimum of 10 events per variable, simulation studies have shown that models with approximately five events per variable can remain stable without substantial overfitting[15]. Therefore, to minimize model instability, only one covariate was included in the adjusted model. The explanatory variables used in the multivariable analysis were the number of days from stoma creation to closure divided by 100, adjusted for the amount of bleeding at the time of stoma closure. Bleeding volume was included in the multivariable model because it reflects both operative complexity and the patient’s postoperative condition, making it a clinically relevant covariate. The results of the logistic regression analysis are reported as unadjusted odds ratios (ORs) for days from stoma creation and 95% confidence intervals (CIs) for the number of days to stoma closure divided by 100 (i.e., the risk of developing infectious enteritis every 100 days) and adjusted OR and 95% CI adjusted for bleeding volume.

## Results

**Figure 1.**
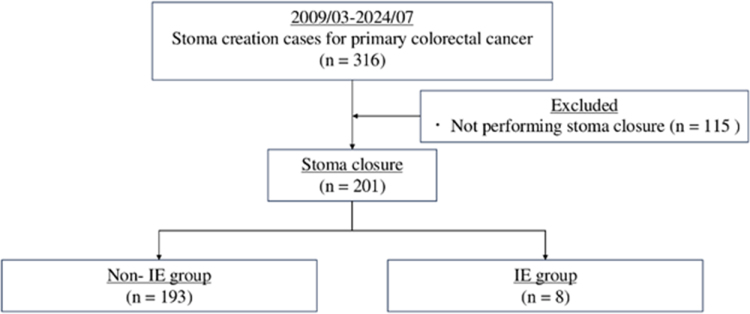
Flowchart of enrolment of study participants. IE, infectious enteritis.

Between March 2009 and July 2024, 316 patients with primary colorectal cancer who underwent surgery with anastomosis and stoma creation at the Department of Gastroenterological Surgery in our hospital were identified. Of these, data from 201 patients who underwent stoma closure were retrospectively reviewed (Fig. 1).

The backgrounds of patients in the IE group and other patients (non-IE group) in this study are shown in Table 1. Of the 201 patients analyzed, eight developed infectious enteritis (CD grade II or higher), whereas 193 did not. Age, sex, BMI, PNI, ASA-PS, smoking history, history of gastrointestinal resection, and primary tumor site did not differ significantly between the IE and non-IE groups.

**Table 1:** Demographic characteristics of the 201 patients who underwent stoma closure for colorectal cancer.

	Non-IE group *n* = 193	IE group *n* = 8	*P*-value
Age (median [IQR])	67 [59, 75]	70 [59, 72]	0.733
Sex (*n*, %)			0.155
Male	117 (60.6)	7 (87.5)	
Female	76 (39.4)	1 (12.5)	
BMI (kg/m^2^) (median [IQR])	22.52 [20.44, 24.57]	21.96 [19.81, 25.40]	0.973
PNI (median [IQR])	48.94 [45.45, 52.55]	49.10 [48.51, 50.79]	0.838
ASA PS (*n*)			1.000
1/2/3	25/160/8	1/7/0	
Smoking (*n*, %)	96 (49.7)	7 (87.5)	0.065
History of gastroenterological surgery (*n*, %)	12 (6.2)	1 (12.5)	1.000
Location of primary tumor site (*n*, %)			0.418
Colon	30 (15.5)	2 (25.0)	
Rectum	163 (84.5)	6 (75.0)	

ASA‑PS, American Society of Anesthesiologists physical status; BMI, body mass index; IE, infectious enteritis; IQR, interquartile range; PNI, prognostic nutritional index.

Information at the time of stoma creation is presented in Table 2. The treatment history before primary tumor location, percentage of rectal resections, percentage of pelvic lymph node dissections, final stage, percentage of complications of CD grade II or higher at the time of primary tumor surgery, percentage of adjuvant chemotherapy between primary surgery and stoma closure, and percentage of treatment history after stoma creation, including neoadjuvant or adjuvant treatment, did not differ significantly between the IE and non-IE groups. A subgroup analysis comparing ileostomy and colostomy (including transverse colostomy) revealed no significant difference in the incidence of infectious enteritis between the stoma types (*P* = 0.847). The results are presented in Supplemental Digital Content Tables 1, available at: http://links.lww.com/MS9/B120.

**Table 2:** Information at the time of stoma creation.

	Non-IE group *n* = 193	IE group *n* = 8	*P*-value
Pre-treatment before primary surgery (*n*, %)	36 (18.7)	3 (37.5)	0.187
Stoma creation before primary surgery (*n*, %)	24 (12.4)	2 (25.0)	0.277
Location of diverting stoma (*n*, %)			1.000
Transverse colon	23 (11.9)	1 (12.5)	
Ileum	170 (88.1)	7 (87.5)	
Rectal resection (*n*, %)	163 (84.5)	6 (75.0)	0.616
Lateral pelvic lymph node dissection (*n*, %)	41 (21.2)	2 (25.0)	0.681
Final stage according to TNM classification (*n*)*			0.491
0/I/II/III/IV	4/67/41/63/18	0/2/4/2/0	
Complication after primary surgery over CD grade II (*n*, %)	65 (33.7)	5 (62.5)	0.130
Adjuvant chemotherapy between primary surgery and stoma closure (*n*, %)	62 (32.1)	4 (50.0)	0.443
Posttreatment after stoma creation (*n*, %)	70 (36.3)	5 (62.5)	0.152

CD, Clavien–Dindo classification; IE, infectious enteritis.

^*^ Cancer stage as determined according to the Union for International Cancer Control tumor node metastasis (TNM) classification of malignant tumors.

Information regarding the time of stoma closure is presented in Table 3. The period from stoma creation to closure was significantly longer, and the volume of bleeding was significantly lower in patients who developed infectious enteritis compared with those who did not (274 vs 114 days, *P* = 0.002; and 0 vs 16 ml, *P* = 0.004). In contrast, the operation time, rate of composite resection, maximum number of bowel movements per day within one week after surgery, duration of perioperative antibiotics administration, rate of postoperative use of probiotics, and number of patients who developed *C. difficile* enteritis after surgery did not differ significantly between the IE and non-IE groups.

**Table 3:** Information at the time of stoma closure.

	Non-IE group *n* = 193	IE group *n* = 8	*P*-value
Period between stoma creation and closure (days, median [IQR])	114 [82, 200]	274 [223, 393]	0.002
Operation time of stoma closure (min, median [IQR])	78 [61, 97]	89 [67, 114]	0.483
Amount of bleeding (ml, median [IQR])	16 [3, 50]	0 [0, 1]	0.004
Composite resection (*n*, %)	13 (6.7)	0 (0.0)	1.000
Postoperative maximum times of stool (times, median [IQR])	7 [4, 13]	9 [5, 13]	0.651
Period of antibiotics administration (days, median [IQR])	1 [1, 1]	1 [1, 1]	0.681
Rate of antiflatulent administration (*n*, %)	169 (87.6)	7 (87.5)	1.000
Number of postoperative *Clostridioides difficile* enteritis (*n*)	0	0	1.000

IE, infectious enteritis; IQR, interquartile range.

**Figure 2.**
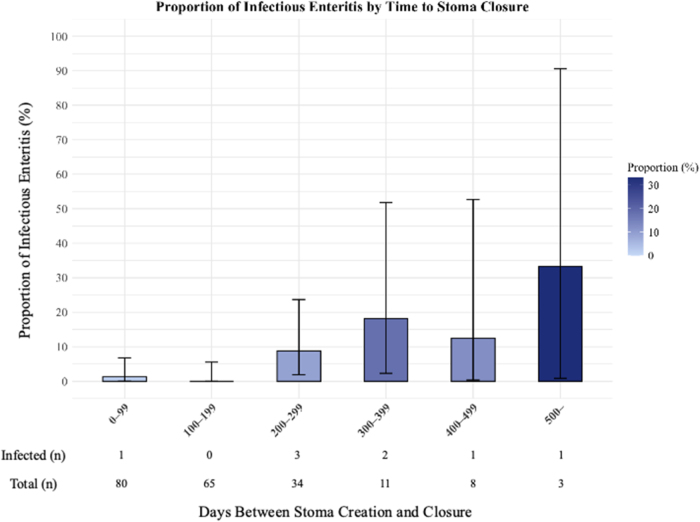
Proportion of infectious enteritis by time to stoma closure. This figure presents descriptive statistics for visualization. Because some bins contained few patients, the total number of cases and events is displayed below the figure. All inferential analyses were performed using the interval as a continuous variable.

Figure 2 shows the incidence of infectious enteritis in 100-day bins until stoma closure. Due to the small number of patients in some bins, this binned plot is presented purely for descriptive visualization. All statistical inferences were made by treating the interval to stoma closure as a continuous variable. In the earliest interval (0–99 days), the incidence of infectious enteritis was approximately 2%. In the ≥500-day bin, one event occurred among three patients, which corresponds to approximately 30%; however, this estimate is based on a very small sample size and should therefore be interpreted with caution. In contrast, no incidence of infectious enteritis was observed in the 100–199-day bin, and the incidence decreased slightly in the 400–499-day bin. The background factors for each group are listed in Supplemental Digital Content Tables 2–7, available at: http://links.lww.com/MS9/B120. In the 200–299-day bin, the median bleeding volumes at stoma closure were 0 and 15 ml in the IE and non-IE groups, respectively (Supplemental Digital Content Tables 4, available at: http://links.lww.com/MS9/B120). The proportions of patients receiving postoperative adjuvant chemotherapy were 0 and 66.7% in the IE group and 32.8 and 77.4% in the non-IE group, respectively, in the 100–199-day and 200–299-day periods, when the rate of postoperative adjuvant chemotherapy is believed to be high (Supplemental Digital Content Tables 3 and 4, available at: http://links.lww.com/MS9/B120).

Table 4 presents the results of the logistic regression analysis of risk factors for the development of infectious enteritis. These results showed that the continuous variable obtained by dividing the number of days from stoma creation to its closure by 100 (i.e., the risk of developing infectious enteritis every 100 days) was an independent predictive factor for the development of infectious enteritis (unadjusted OR: 1.574, 95% CI: 1.138–2.411, *P* = 0.025). Furthermore, even after adjusting for the amount of bleeding, the number of days from stoma creation to its closure tended to contribute to the incidence of infectious enteritis (adjusted OR: 2.949, 95% CI: 1.492–6.838, *P* = 0.005).

**Table 4:** Logistic regression analysis on the incidence of infectious enteritis.

Variable	Odds ratio (unadjusted)	95% confidence interval (unadjusted)	*P*-value (unadjusted)	Odds ratio (adjusted)	95% confidence interval (adjusted)	*P*-value (adjusted)
(Period between stoma creation and closure)/100	1.574	1.138–2.411	0.025	2.949	1.492–6.838	0.005
Bleeding				0.688	0.376–0.897	0.090

## Discussion

In this study, we identified the risk factors that contribute to the development of infectious enteritis during hospital stays after stoma closure in patients with a temporary stoma following surgery for colorectal cancer. Although infectious enteritis often occurs after stoma closure, previous large-scale cohort studies did not examine this complication. To our knowledge, this study is the first to report risk factors for infectious enteritis in patients who have undergone stoma closure.

In our cohort, the time to closure was significantly longer, and the amount of bleeding was lower in the group that developed infectious enteritis during the hospital stay than in the group that did not. In addition, by dividing the period until the stoma closure into 100-day periods, we demonstrated the increasing incidence of infectious enteritis as the period increased. Furthermore, the results of the multivariable analysis showed that the period until stoma closure, divided into 100-day periods, was an independent predictor of the onset of infectious enteritis. This result is consistent with previous findings of increased complications and *C. difficile* infection with a longer time to closure^[^6,16,17^]^. A previous review did not report on the significant difference between the amount of bleeding and complications after stoma closure[18]. Although postoperative complications are reportedly associated with high bleeding volumes after colorectal cancer surgery^[^19,20^]^, no associations between small bleeding volumes and complications have been reported. In this study, an inverse association between bleeding volume and the development of infectious enteritis was observed. Because the bleeding volume during stoma closure was generally very small, this relationship should be interpreted with caution. Postoperative adhesions initially form in the context of acute inflammation and neovascularization, and early adhesions are characterized as highly vascular and friable, which increases the likelihood of bleeding during dissection[21]. As adhesions mature over time, their tissue characteristics change, and differences in the timing of stoma closure may have contributed to the observed variation in bleeding volume. Given the small number of infectious enteritis events, random variation and residual confounding may also have influenced the association. Therefore, this finding should be regarded as hypothesis-generating and not interpreted as causal.

One consideration that supports the results of this study is the effect of changes in the intestinal tract on the anal side of the stoma, where intestinal fluid does not pass for long periods. In patients with a long period until stoma closure, the composition of the intestinal microbiota on the anal side of the stoma changes, affecting the intestinal tract structure and function[22]. Similarly, diarrhea symptoms accompanied by bacterial translocation after anastomotic closure may occur because of these changes in the intestinal tract^[^23,24^]^.

Therefore, treatment methods for patients with a long-term stoma should be considered. Whether intestinal fluid originates from the oral side of the intestinal tract, as well as the use of prebiotics, probiotics, or symbiotics, remains controversial. A randomized controlled trial (RCT) on the use or non-use of probiotics after stoma closure did not observe improved effectiveness in terms of recovery of intestinal function after surgery; however, this RCT did not consider the onset of enteritis[25]. Additionally, although complications after anastomosis are reportedly reduced by injecting intestinal fluid into the anal side of the stoma in infants (refeeding)^[^26,27^]^ other reports demonstrated increased incidences of complications[26] and increased levels of skin commensal and pathogenic bacteria with longer excrement duration in the stoma bag[28]. Further large-scale studies in adults are needed to determine the effectiveness of refeeding and its potential effectiveness in preventing complications. In addition, an RCT is currently underway, in which probiotics are administered via the anus before stoma closure[29].

Several factors can prolong the time to stoma closure, most notably postoperative treatments administered after stoma creation and complications during surgery for the primary lesion. In our cohort, a substantial proportion of patients underwent postoperative therapies, including adjuvant chemotherapy and total neoadjuvant therapy, which resulted in delayed closure. Nevertheless, preoperative treatment, postoperative adjuvant chemotherapy, preoperative total neoadjuvant therapy, and preoperative chemoradiotherapy were not associated with the development of infectious enteritis. These findings are consistent with a previous meta-analysis demonstrating similar complication rates between stoma closures performed during adjuvant chemotherapy and those completed after treatment[30]. The results of a prospective multicenter RCT currently underway in Italy to determine whether it is better to close the stoma before or after adjuvant chemotherapy in patients eligible for postoperative adjuvant chemotherapy are greatly anticipated[31]. Although closing the stoma too early without care may increase the risk of suture failure[32], early stoma closure is also associated with significantly fewer complications than late closure in patients with temporary stoma after rectal cancer surgery with no clinical or radiological signs of suture failure[33]. Therefore, the timing of stoma closure must also be considered from the perspective of the risk of developing infectious enteritis.

Regarding complications during surgery for the primary lesion, although these did not appear to be associated with the onset of infectious enteritis, as complications during surgery for the primary lesion can delay postoperative chemotherapy initiation, additional data should be collected. Additionally, as many other factors can affect the time to closure, future studies should also examine these factors in detail, from stoma creation to closure.

Our study has some limitations. First, this was a single-center retrospective study. Our findings indicate the need for future studies that provide additional validation and address these limitations, including multicenter and registry studies. Second, because the study covers an extended time span, changes in perioperative management and diagnostic practices over time may have introduced heterogeneity. This temporal variability is an unavoidable limitation of retrospective studies. Third, the reasons for delayed stoma closure were not extracted for all cases. Although the clear cut-off value for the delay is unclear, clarifying the reasons for the delay may prevent unnecessarily delayed closure. Fourth, the number of infectious enteritis events was small, which limits statistical power and increases the potential for random variation. As a result, some associations, such as those involving bleeding volume, should be interpreted with caution. Additionally, detailed microbiological data were not available for most cases because comprehensive stool testing is not routinely performed after rectal cancer surgery. Postoperative changes in bowel habits and inflammatory responses often limit the interpretability of stool cultures, and testing is generally reserved for suspected *C. difficile* infection. Consequently, the diagnosis of infectious enteritis in this study relied primarily on clinical symptoms, inflammatory markers, and imaging findings rather than pathogen confirmation, and this lack of microbiological specificity represents a notable limitation. Finally, standardized diagnostic criteria for infectious enteritis are lacking; therefore, some degree of misclassification cannot be ruled out.

The results of this study emphasize the need to elucidate the risk factors for the development of infectious enteritis after stoma closure using detailed information from ileostomy creation to closure and for research into prevention and treatment for cases with increased times to closure. Decisions regarding the timing of stoma closure should therefore continue to consider oncologic treatment requirements, anastomotic safety, and the patient’s overall clinical condition.

## Data Availability

The datasets used and/or analyzed during the current study are available from the corresponding author on reasonable request.

## Research registration unique identifying number (UIN)

The study protocol was approved by the Ethics Committee of Yokohama City University Hospital (approval number: F240700003).

## Assistance with the study

We thank Editage (www.editage.com) for editing our manuscript.
